# Single-Dose, Intranasal Immunization with Recombinant Parainfluenza Virus 5 Expressing Middle East Respiratory Syndrome Coronavirus (MERS-CoV) Spike Protein Protects Mice from Fatal MERS-CoV Infection

**DOI:** 10.1128/mBio.00554-20

**Published:** 2020-04-07

**Authors:** Kun Li, Zhuo Li, Christine Wohlford-Lenane, David K. Meyerholz, Rudragouda Channappanavar, Dong An, Stanley Perlman, Paul B. McCray, Biao He

**Affiliations:** a Department of Pediatrics, Pappajohn Biomedical Institute, University of Iowa, Iowa City, Iowa, USA; b Department of Pathology, University of Iowa, Iowa City, Iowa, USA; c Department of Microbiology and Immunology, University of Iowa, Iowa City, Iowa, USA; d Department of Infectious Diseases, College of Veterinary Medicine, University of Georgia, Athens, Georgia, USA; The Peter Doherty Institute for Infection and Immunity

**Keywords:** COVID-19, MERS, coronavirus, vaccine

## Abstract

Middle East respiratory syndrome coronavirus (MERS-CoV) can cause severe and fatal acute respiratory disease in humans and remains endemic in the Middle East since first being identified in 2012. There are currently no approved vaccines or therapies available for MERS-CoV. In this study, we evaluated parainfluenza virus 5 (PIV5)-based vaccine expressing the MERS-CoV envelope spike protein (PIV5/MERS-S) in a human DPP4 knockin C57BL/6 congenic mouse model (hDPP4 KI). Following a single-dose intranasal immunization, PIV5-MERS-S induced neutralizing antibody and robust T cell responses in hDPP4 KI mice. A single intranasal administration of 10^4^ PFU PIV5-MERS-S provided complete protection against a lethal challenge with mouse-adapted MERS-CoV (MERS_MA_6.1.2) and improved virus clearance in the lung. In comparison, single-dose intramuscular immunization with 10^6^ PFU UV-inactivated MERS_MA_6.1.2 mixed with Imject alum provided protection to only 25% of immunized mice. Intriguingly, an influx of eosinophils was observed only in the lungs of mice immunized with inactivated MERS-CoV, suggestive of a hypersensitivity-type response. Overall, our study indicated that PIV5-MERS-S is a promising effective vaccine candidate against MERS-CoV infection.

## Abstract (precis)

MERS-CoV causes lethal infection in humans, and there is no vaccine. Our work demonstrates that PIV5 is a promising vector for developing a MERS vaccine. Furthermore, success of PIV5-based MERS vaccine can be employed to develop a vaccine for emerging CoVs such as SARS-CoV-2, which causes COVID-19.

## INTRODUCTION

Middle East respiratory syndrome (MERS) emerged as a significant illness on the Saudi Arabian peninsula in mid-2012, and the causative agent was identified as a novel coronavirus (CoV), MERS-CoV (1). MERS has a high mortality rate (∼35%) associated with severe lung disease that can advance to acute respiratory distress syndrome (ARDS). MERS-CoV, similarly to SARS-CoV, which caused a similar epidemic in 2003, has been a global cause for concern due to its high fatality rate. Epidemiologic studies established that MERS-CoV is zoonotic in origin, with transmission occurring from dromedary camels on the Arabian peninsula (2–4). Spread from camels to people is documented (5), as well as person-to-person spread among health care workers in hospital settings (6). To date, MERS-CoV has spread to 27 countries and caused 858 deaths in 2,494 confirmed cases (4 February 2020, World Health Organization [WHO]), including a large travel-related outbreak in South Korea in 2015 (7).

MERS-CoV is an enveloped positive-stranded RNA virus whose entry into target cells is mediated by the viral envelope S protein. The S protein consists of an S1 subunit responsible for binding to the virus receptor, dipeptidyl peptidase 4 (DPP4 or CD26), via a receptor-binding domain (RBD), and an S2 subunit that mediates membrane fusion (8–10). Thus, the S protein, particularly the RBD, is an important target for MERS-CoV vaccine development (8, 11, 12). There is currently no vaccine or antiviral therapeutic against MERS-CoV. A number of candidate MERS-CoV vaccines, including those based on recombinant virus, viral vectors (e.g., MVA, adenovirus, and measles virus), nanoparticles, DNA, and DNA/protein, as well as subunit vaccines, are under development (12, 13). None are approved; thus, the need remains for an effective and broad-spectrum vaccine against MERS-CoV infection (14).

PIV5, formerly known as simian virus 5 (SV5), is a nonsegmented, negative-strand, RNA virus (NNSV). It is a member of the *Rubulavirus* genus of the family *Paramyxoviridae*, which includes mumps virus (MuV) and human parainfluenza virus type 2 (HPIV2) and type 4 (HPIV4) (15). PIV5 encodes eight known viral proteins (15). Nucleocapsid protein (NP), phosphoprotein (P), and large RNA polymerase (L) protein are important for transcription and replication of the viral RNA genome. PIV5 is an excellent viral vector candidate for vaccine development; it is safe and infects a large number of mammals without being associated with any diseases, except kennel cough in dogs (16–20). Because PIV5 does not have a DNA phase in its life cycle, its use avoids the possible unintended consequences of genetic modifications of host cell DNA through recombination or insertion. In comparison to positive-strand RNA viruses, the genome structure of PIV5 is stable. A recombinant PIV5 expressing F of respiratory syncytial virus (RSV) has been generated, and the F gene was maintained for more than 10 generations (21). PIV5 can be grown to 8 × 10^8^ PFU/ml, indicating its potential as a cost-effective and safe vaccine vector that may be used in mass production. We have discovered that PIV5-based influenza, respiratory syncytial virus (RSV), and rabies vaccines are efficacious (22–28). In studies of influenza, we previously reported that that a PIV5 vector expressing influenza virus NA provided sterilizing immunity (no mortality, no morbidity, and no virus detected in the lungs of challenged mice at 4 days postchallenge) and PIV5 expressing NP protected 100% of mice against lethal influenza virus H1N1 challenge in mice (25), demonstrating that PIV5 is an excellent vector for developing vaccines for respiratory pathogens. Here we investigate the utility of a PIV5-based vaccine expressing the MERS S protein in a robust humanized mouse model of lethal MERS-CoV infection.

## RESULTS

### Construction of a PIV5 vector expressing MERS-CoV spike glycoprotein.

**FIG 1:**
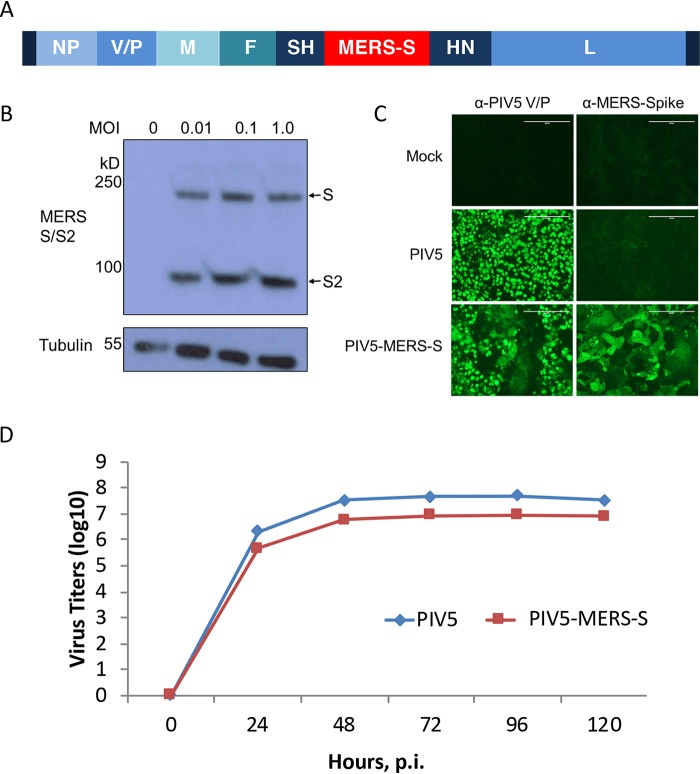
Generation and characterization of recombinant PIV5 expressing MERS-CoV spike protein. (A) Schematic of PIV5-MERS-S. NP, nucleoprotein; V, V protein; P, phosphoprotein; M, matrix protein; F, fusion protein; SH, small hydrophobic protein; HN, hemagglutinin-neuraminidase protein; L, RNA-dependent RNA polymerase. (B) Confirmation of MERS-CoV spike protein expression by Western blotting. Vero 81 cells were infected with PIV5-MERS-S at MOIs of 0.01, 0.1, and 1.0 or mock infected. At 2 days postinfection, MERS-CoV spike was detected with anti-MERS-S antibody by Western blotting. (C) Immunofluorescence of Vero cells infected with PIV5 and PIV5-MERS-S. Vero cells were infected with PIV5 and PIV5-MERS-S (MOI = 0.1) or mock infected. At 2 days postinfection, cells were fixed, permeabilized, and stained with anti-PIV5 V/P or anti-MERS-spike antibodies. Scale bar = 200 μm. (D) Growth rate of PIV5-MERS-S. MDBK cells were infected with PIV5 or PIV5-MERS-S at an MOI of 0.1. Media were collected daily for 5 days, and titers of viruses in the media were determined using plaque assay.

Previously, we inserted the HA gene of influenza A virus at different locations within the genome of PIV5 and found that the insertion at SH and HN generates the best immune responses (24). Thus, we inserted the full-length gene of S of MERS at the SH and HN junction. A plasmid containing full-length PIV5 cDNA with the S gene insertion at SH and HN junction was constructed using standard molecular cloning techniques (Fig. 1A). The plasmid was transfected into BHK cells along with plasmids expressing T7 RNA polymerase, NP, P, and L of PIV5, and infectious virus PIV5-MERS-S was rescued as described before (24). The rescued virus was plaque-purified and then expanded to large quantity in MDBK cells for further analysis. The viral genome was sequenced and confirmed to contain the desired input DNA sequence. To verify S protein expression in PIV5-MERS-S-infected cells, the cells were infected at different MOIs and then lysed for immunoblotting using anti-S antibody. The full-length S and cleaved S2 fragments were observed in PIV5-MERS-S-infected cells, suggesting that the S protein was properly processed (Fig. 1B). Expression of S protein in PIV5-MERS-S-infected cells was further confirmed by immunofluorescence assay (Fig. 1C). Interestingly, PIV5-MERS-S caused massive syncytium formation in Vero cells. PIV5-MERS-S had a similar growth kinetics as wild-type PIV5 (Fig. 1D).

### Immunization with PIV5 MERS-S generates neutralizing antibodies and T cell-mediated immunity.

**FIG 2:**
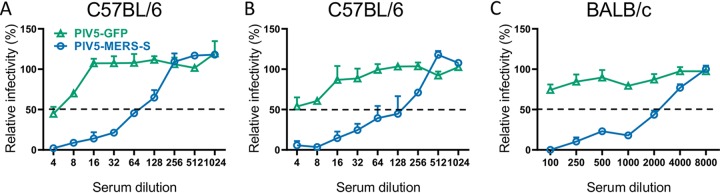
Serum neutralizing antibodies produced in mice 4 weeks after single-dose intranasal immunization with PIV5-MERS-S. Naive mice were intranasally immunized with PIV5-GFP or PIV5-MERS-S. Sera were collected at 4 weeks postimmunization. Neutralization assay against MERS-CoV spike pseudovirions was performed as described in Materials and Methods. The neutralization results were measured in luciferase activity and plotted relative to mock-treatment value. (A and B) Neutralization assay results from C57BL/6 mice immunized with 10^4^ PFU (A) and 10^6^ PFU (B) PIV5-MERS-S or PIV5-GFP. (C) Neutralization assay results from BALB/c mice immunized with 10^6^ PFU PIV5-MERS-S or PIV5-GFP. Data presented represent means ± SEs.

To determine whether PIV5-MERS-S can generate immune responses in mice, C57BL/6 mice were immunized with a single dose of PIV5-MERS-S or control PIV5-GFP virus at 10^4^ PFU or 10^6^ PFU per mouse via intranasal route. While both doses generated antibody responses, neutralizing titers were modest at 1:64 and 1:128 for the 10^4^ and 10^6^ doses, respectively (Fig. 2A and B). It is known that C57BL/6 and BALB/c mice generate Th1- and Th2-dominant immune responses, respectively, following immunization. A single dose of 10^6^ PFU of PIV5-MERS-S resulted in a neutralization antibody titer as high as 1:2,000 in BALB/c mice (Fig. 2C), consistent with a Th2-dominant response in BALB/c mice.

**FIG 3:**
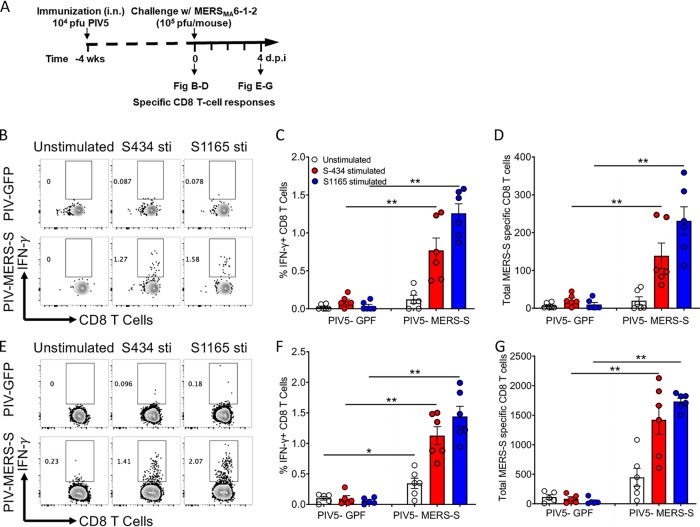
Single-dose intranasal immunization with PIV5-MERS-S induced robust MERS-CoV-specific CD8 T cell response in human DPP4 knockin (hDPP4 KI) mice. (A) Schematic diagram showing the experimental plan to examine CD8 T cell response after immunization and challenge. hDPP4 KI mice were intranasally immunized with 10^4^ PFU PIV5-MERS-S or PIV5-GFP. At 4 weeks or 4 days following immunization, mice were challenged with MERS_MA_6.1.2, single-cell suspensions from the lungs of immunized mice were stimulated with MERS-CoV spike peptides (S343 and S1165), and specific CD8 T cells were determined by IFN-γ intracellular staining. (B to D) Representative FACS plots (B), percentage (C), and total number (D) of MERS-CoV-specific CD8 T cells in the lungs at 4 weeks after immunization. (E to G) Representative FACS plots (E), percentage (F), and total number (G) of MERS-CoV-specific CD8 T cells in the lungs at 4 days after MERS_MA_6.1.2 challenge (*n* = 6 mice per group). Data are representative of two independent experiments. Data presented represent mean ± SE; * denotes *P* < 0.05 and ** denotes *P* < 0.01, Mann-Whitney test.

To assess the primary CD8 T cell response generated by PIV5-MERS-S immunization, hDPP4-KI mice were intranasally immunized with 10^4^ PFU PIV5-MERS-S. Four weeks later, lungs were harvested and examined for MERS-CoV-specific lung-resident CD8 T cells (Fig. 3A). As shown in Fig. 3B to D, we observed a significant increase in the percentage and total number of CD8^+^-IFN-γ^+^ cells in the lungs of PIV5-MERS-S-immunized mice in comparison to those infected with PIV5-GFP virus, consistent with a MERS-S-specific primary CD8 T cell response in the lungs. Further, to examine recall response of MERS-S-specific CD8 T cells, we challenged PIV5-MERS-S- and PIV5-GFP-immunized mice with 10^4^ PFU of MERS-CoV_MA_6.1.2. Our results show a significant increase in the recall CD8 T cell response at day 4 p.i. in comparison to PIV5-GFP-immunized mice (Fig. 3E to G). We also observed 10-fold increase in MERS-S-specific CD8 T cells compared to the primary CD8 T cell response (Fig. 3B to G). Collectively, these results indicate that MERS-S immunization induces a significantly increased MERS-S-specific CD8 T cell response upon PIV5-MERS-S immunization.

### Immunization with PIV5-MERS-S prevents lethal infection in mice.

**FIG 4:**
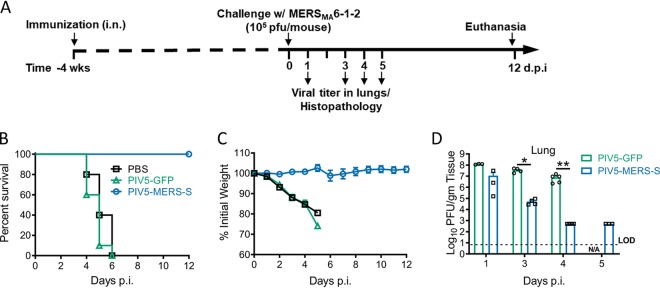
Single-dose intranasal immunization with PIV5-MERS-S completely protects hDPP4 KI mice from lethal MERS-CoV challenge. (A) Schematic timeline showing immunization, challenge, and the evaluation of protection. hDPP4 KI mice were intranasally immunized with 10^4^ PFU PIV5-MERS-S, PIV5-GFP, or PBS. Four weeks after immunization, the mice were intranasally infected with 10^5^ PFU MERS_MA_6.1.2. (B and C) Survival (B) and weight loss (C) were monitored daily for 12 days. PIV5-MERS-S or PIV5-GFP, *n* = 10; PBS, *n* = 5. (D) At indicated days postinfection, virus lung titers were quantified by plaque assay. Data are representative of two independent experiments. Data presented represent mean ± SE; * denotes *P* < 0.05 and ** denotes *P* < 0.01, Mann-Whitney test. LOD, limit of detection.

To determine the efficacy of PIV5-MERS-S in preventing or modifying a MERS-CoV infection, hDPP4 KI mice on the C57BL/6 background were immunized with 10^4^ PFU PIV5-MERS-S via the intranasal route. At 4 weeks after immunization, mice were challenged with a mouse-adapted MERS-S strain (Fig. 4A). All PIV5-MERS-S-immunized mice survived this lethal challenge and lost little weight (Fig. 4B and C). In contrast, PIV5-GFP- or PBS-immunized mice all died following challenge (Fig. 4B and C), indicating that PIV5-MERS-S completely protected mice against lethal challenge. While PIV5-MERS-S-immunized mice had a higher rate of virus clearance from the lungs, this did not provide sterilizing immunity (Fig. 4D).

### Histopathology of lung tissues.

**FIG 5:**
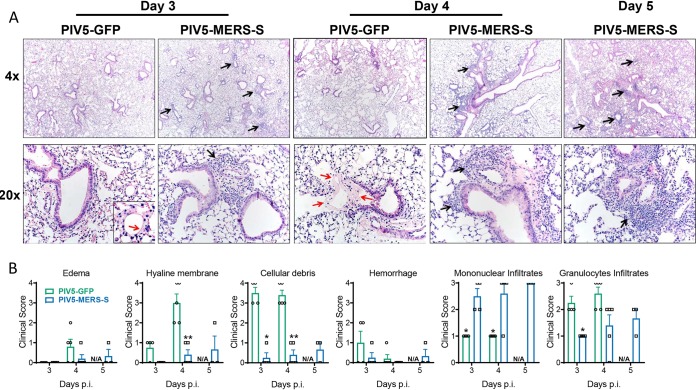
Histopathology in immunized mice challenged with MERS-CoV. hDPP4 KI mice were intranasally immunized with 10^4^ PFU PIV5-MERS-S or PIV5-GFP. Four weeks after immunization, the mice were intranasally infected with 10^5^ PFU MERS_MA_6.1.2. (A) Representative images of H&E-stained lung sections from PIV5-MERS-S- or PIV5-GFP-immunized hDPP4 KI mice at indicated days after MERS_MA_6.1.2 challenge. Note the cellular infiltration (black arrows) and the hyaline membranes (red arrows). (B) Summary scores for disease in the lung sections. *n* = 3 to 5 mice/group. * denotes *P* < 0.05, Mann-Whitney test; N/A denotes not applicable.

Histopathology studies of lungs after challenge with MERS_MA_6.1.2 indicated that PIV5-MERS-S-immunized mice had significantly less cellular debris present and greater mononuclear infiltrates (Fig. 5A and B). PIV5-MERS-S-immunized mice exhibited robust cellular infiltration of leukocytes (mostly mononuclear cells) and less evidence of lesions (edema, hyaline membranes, necrotic cellular debris, etc.) indicative of severe disease (Fig. 5).

### Comparison of PIV5-MERS-S to inactivated MERS-CoV.

**FIG 6:**
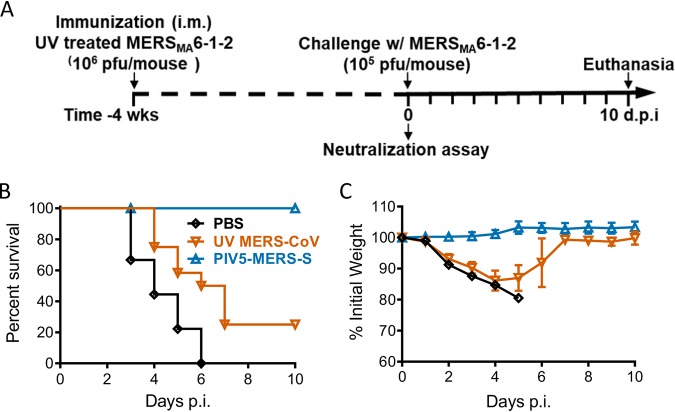
Comparison of the protective efficacy between single-dose immunization with UV light-inactivated MERS-CoV and PIV5-MERS-S. hDPP4 KI mice were immunized with 10^4^ PFU PIV5-MERS-S via intranasal route; 10^6^ PFU UV-inactivated MERS_MA_6.1.2, mixed with Imject alum; or PBS via intramuscular route. Four weeks after immunization, immunized mice were infected with 10^5^ PFU MERS-CoV. (A) Schematic timeline outlining experimental plan. (B and C) Survival (B) and weight loss (C) were monitored daily until 10 days postinfection. PBS, *n* = 9; UV MERS-CoV, *n* = 12; PIV5-MERS-S, *n* = 8. Data represent mean ± SE.

To investigate the protective responses elicited by PIV5-MERS-S and the inactivated MERS-CoV, hDPP4 KI mice were immunized with 10^4^ PFU of PIV5-MERS-S or PBS via i.n. or UV-inactivated MERS-CoV with adjuvant via i.m. route. While PIV5-MERS-S provided 100% protection against lethal challenge, inactivated MERS-CoV protected 25% of mice from mortality (Fig. 6).

**FIG 7:**
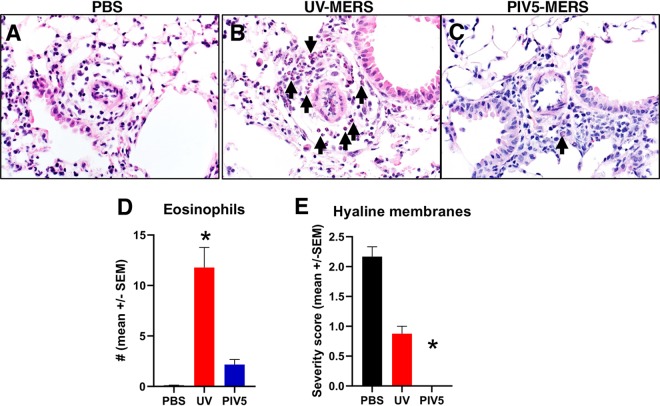
Representative images of lung tissues from mice receiving PBS (A), UV-inactivated MERS-CoV (B), or PIV5-MERS-S (C) treatment, followed by infection with MERS_MA_6.1.2. Images obtained from tissues at 3 days after MERS_MA_6.1.2 infection. Compared to PBS or PIV5-MERS, perivascular eosinophilic infiltration (arrows) in UV-MERS-treated mice was greatly increased. *n* = 3 to 4 mice/group. (D) Graph representing eosinophil infiltration in the lung tissues of mice from groups shown in panels A to . * denotes *P* < 0.009, Dunn’s posttest. Other group comparisons were not significant. (E) Graph representing hyaline membranes in the lung tissues of mice from groups shown in panels A to C. * denotes *P* < 0.0065, Dunn’s posttest. Other group comparisons were not significant.

It has been reported that mice immunized with inactivated SARS-CoV and MERS-CoV developed a hypersensitivity-type response after respective SARS-CoV and MERS-CoV challenge, manifested by increased IL-4 and IL-5 expression and an influx of eosinophils (29). We examined the lungs of mice that were immunized and then challenged with MERS-CoV (Fig. 7). We observed more eosinophils in the lungs of mice immunized with inactivated MERS-CoV than in PBS- or PIV5-MERS-S-immunized mice following MERS-CoV challenge. Compared to PBS, perivascular eosinophilic infiltration was significantly increased in the inactivated MERS-CoV group, but no statistical difference was seen when compared with the PIV5-MERS-S-immunized group. In these same lung tissues, we evaluated hyaline membrane formation as a measure of diffuse alveolar damage. Compared to the PBS group, the PIV5-MERS-S-immunized mice demonstrated significant protection against hyaline membrane formation, while the inactivated MERS-CoV group had only minor nonsignificant reductions in hyaline membrane formation. These results suggest that inactivated MERS-CoV may have caused a hypersensitivity-type response, while PIV5-MERS-S-immunized mice had minimal lung influx of eosinophils and were protected.

## DISCUSSION

Many strategies have been considered to develop vaccines for both SARS-CoV and MERS-CoV. A live attenuated SARS-CoV with rationally introduced mutations was efficacious in golden Syrian hamsters (30). However, the development of a live attenuated vaccine for a positive-stranded RNA virus like SARS-CoV has often been hampered by safety concerns. Several MERS vaccine candidates are under investigation. A DNA-based vaccine expressing the full-length S protein is the most advanced to date (31); it is well tolerated in humans, as shown in a phase I clinical trial. The prime-boost regimen of MVA (Modified Vaccinia Ankara) expressing MERS S protein induced neutralizing antibodies and T cell responses in mice and limited viral replication after challenge in mice and camels. However, MVA-S did not provide sterilizing immunity, and infectious MERS-CoV and genomic RNA were detected after challenge in mice and camels (32, 33). The prime-boost regimen of measles virus (MV) expressing MERS S or soluble S induced both humoral and cellular immune responses. After MERS challenge, infectious MERS-CoV or genomic RNA significantly decreased, but these two vaccines did not provide sterilizing immunity, and signs of inflammation were observed in mouse lung tissue (34). An inactivated rabies virus (RABV) expressing MERS S1 provided complete protection from MERS challenge in mice but three 10-μg doses of vaccine were needed (35). Furthermore, the Ad5/hDPP4-transduced mouse model used in these studies has limitations. Adenovirus (Ad5) expressing MERS S or S1 also induced a neutralizing antibody in mice (36). Ad41, an enteric adenovirus, may induce enhanced mucosal immunity when administered via an oral or intragastric (i.g.) route (37, 38). However, i.g. immunization of both Ad41-S and Ad5-S failed to generate mucosal immunity. Although Ad41-S induced humoral immunity in serum, it was significantly less than Ad5-S (39). Chimpanzee adenovirus-based vector systems have also been used (40). In our work, we demonstrated that a single dose as low as 10^4^ PFU of PIV5-MERS-S was sufficient to provide 100% protection against lethal MERS-CoV challenge. The low dose is especially advantageous in a situation where a mass immunization program is needed in a short period of time. To the best of our knowledge, this is the most efficacious MERS-CoV vaccine tested in a relevant animal model.

The protective mechanism of PIV5-MERS-S vaccine in C57BL/6 mice is likely due to robust cellular immune responses after PIV5-MERS-S immunization. While neutralizing antibody was generated in C57BL/6 mice after a single-dose immunization with PIV5-MERS-S, titers were modest at 1:64 and 1:128 with 10^4^ PFU and 10^6^ PFU of PIV5-MERS-S, respectively (Fig. 2A and B). Consistent with protective cellular immune responses protecting the mice, a significant influx of CD8^+^ IFN-γ^+^ cells was detected in lungs of C57BL/6 mice following PIV5-MERS-S immunization (Fig. 3). Furthermore, the observation that PIV5-MERS-S-immunized mice had a higher rate of MERS virus clearance (Fig. 4C) suggests a role for T cell-based immunity in protecting C57BL/6 mice against lethal challenge. Interestingly, in BALB/c mice, PIV5-MERS-S generated neutralizing antibody titers as high as 1:2,000 (Fig. 2C). It is possible that the higher neutralizing antibody titers in BALB/c mice may be protective. Unfortunately, the only available small animal model is a humanized mouse model on the C57BL/6 background. It is known that the S protein is a major protective antigen for coronaviruses. It may be possible to improve our vaccine efficacy by expressing additional MERS-CoV proteins such as N and M using PIV5 as a vector. However, a parainfluenza virus 3 (PIV3)-based SARS-CoV vaccine candidate expressing N, M, or E without the S protein failed to protect hamsters from SARS-CoV challenge (41). The ability of PIV5-MERS-S to generate cellular and humoral immune responses in mice may be in part attributed to the ability of PIV5 to express the MERS S protein in its native conformation. As shown in Fig. 1C, PIV5-MERS-S caused massive syncytium formation in Vero cells, indicating the S protein was functional in promoting cell-to-cell fusion. Thus, we reasoned that the S protein produced in PIV5-MERS-S-infected cells maintains a native conformation.

The MERS S protein has 1,353 amino acid residues. The entire insertion of the S gene with proper regulatory sequences is over 4,000 nucleotides in length. This is the longest single gene we have inserted into the PIV5 genome. Since we inserted this gene between SH and HN, and the SH gene is not essential, it may be possible to remove SH to allow insertion of longer sequences. Thus, we speculate that the PIV5 genome can accommodate sequences longer than 4,000 nucleotides.

It has been reported that inactivated SARS-CoV-immunized mice generated a hypersensitive-type lung pathology after virus challenge, raising the concern of vaccine-enhanced disease (42, 43). Previously, a formalin-inactivated, whole-virus respiratory syncytial virus (RSV) vaccine caused enhanced disease in vaccinated children, leading to vaccine-related deaths (44). Similarly, inactivated MERS-CoV has been reported to generate a Th2-type immunopathology after MERS-CoV challenge in mice (29). In the case of a PIV5-based RSV vaccine, extensive studies indicate that PIV5-based RSV vaccine does not cause enhanced diseases (45). Thus, as a viral vector, PIV5 is not known to cause any enhanced diseases, and in our experiment, we observed no abnormal immune responses in PIV5-MERS-S-immunized mice after MERS-CoV challenge, suggesting that PIV5-MERS-S is unlikely to be associated with enhanced disease. Lung tissues of mice immunized with inactivated MERS-CoV had an influx of eosinophils after MERS-CoV challenge, indicative of a hypersensitivity-type response. This result is consistent with a previous report that inactivated MERS-CoV immunization caused increased IL-4 and IL-5 expression and an influx of eosinophils in lungs after challenge (29). Understanding whether immunization with inactivated MERS-CoV can cause enhanced disease is critical for developing a safe and effective vaccine.

While MERS-CoV has a high morbidity and mortality, it has very a low prevalence in human populations. Dromedary camels are considered the intermediate host that transmits MERS-CoV to humans. Thus, it may be possible to control the spread of MERS-CoV in humans by controlling infection in dromedary camels. Perhaps virus transmission from camels to humans can be blocked, with concomitant immunization of high-risk human populations, as proposed by CEPI (The Coalition for Epidemic Preparedness Innovations) and WHO. As a vaccine vector, PIV5 has been effective in mice, cotton rats, hamsters, guinea pigs, ferrets, dogs, and nonhuman primates (25, 46–49). It will be worthwhile to test PIV5-MERS-S in camels in the future.

Recently, SARS-CoV-2 (2019-nCoV) was identified in Wuhan, China, in late 2019. This is a novel zoonotic CoV related to the SARS-CoV that can cause severe respiratory disease (COVID-19). To date, this virus resulted in a significant disease burden, with more than 465,000 cases reported in 199 countries and an estimated case fatality rate of ~2%. The finding that PIV5 expressing MERS S protected mice against lethal MERS-CoV challenge at a single low dose of 10^4^ PFU suggests its potential use as a vaccine vector for emerging viruses such as SARS-CoV-2. Further studies of using PIV5 expressing the S protein from SARS-CoV-2 as a vaccine candidate are ongoing.

## MATERIALS AND METHODS

### Cells.

Vero cells were maintained in Dulbecco’s modified Eagle medium (DMEM) supplemented with 10% fetal bovine serum (FBS), 100 IU/ml penicillin, and 100 μg/ml streptomycin (1% P/S; Mediatech Inc., Manassas, VA, USA). BHK21 cells were maintained in DMEM containing 10% tryptose phosphate broth (TPB), 10% fetal bovine serum (FBS), 100 IU/ml penicillin, and 100 μg/ml streptomycin. MDBK cells were grown in DMEM containing 5% FBS and 1% P/S. Cells were prepared 1 day prior to infection, achieving approximately 90% confluence by the following day.

### Viruses.

The plasmid containing the cDNA clone of PIV5 with MERS-S inserted between SH and HN was constructed using previously described methods (22, 26, 29). Primer sequences are available upon request. Infectious virus was rescued in BHK cells as previously described (26). Recombinant PIV5 or PIV5-MERS-S was propagated in MDBK cells as previously described (26, 29).

PIV5 plaque assays were performed as previously described (24). Briefly, 10-fold serial dilutions were prepared in DMEM with 1% BSA. One hundred microliters of each dilution was transferred to 6-well plates of BHK21 cells, in a total infection volume of 1 ml. After adsorption for 1 to 2 h at 37°C, 5% CO_2_, the inocula were aspirated, and cell monolayers were overlaid with DMEM containing 10% tryptose phosphate broth (TPB), 2% FBS, 1% P/S, and 1% low-melting-point agarose. After 5 days, the cells were fixed with 2% formaldehyde, overlays were removed, and the cells were stained with crystal violet to visualize the plaques.

To obtain virus titers in lung tissues, lungs of infected mice were removed at the indicated days after challenge and homogenized in PBS using a manual homogenizer. Virus titer was determined in Vero 81 cells by plaque assay. Infected Vero 81 cells were fixed in 25% formaldehyde and stained with 0.1% crystal violet to delineate plaques.

To determine growth rates of PIV5 and PIV5-MERS-S, MDBK cells were infected with PIV5 or PIV5-MERS-S at an MOI of 0.1. After adsorption for 1 to 2 h at 37°C, 5% CO_2_, DMEM with 2% FBS and 1% P/S was added to the plates. One-hundred-microliter samples of supernatant were collected daily for 5 days and frozen at −80°C. Virus titers in the samples were quantified by plaque assay.

### Immunization and infection of mice.

Specific-pathogen-free 6-week-old C57BL/6 and BALB/c mice were purchased from Charles River Laboratory (CR). Specific-pathogen-free human DPP4 knockin (hDPP4 KI) mice were generated on a C57BL/6 background as previously reported (50). All mice were bred and maintained in the University of Iowa animal care facility. All protocols were reviewed and approved by the University of Iowa Institute Animal Care and Use Committee. Six- to 8-week-old male and female mice were used for these studies. Mouse-adapted MERS-CoV strain MERS_MA_6.1.2 was generated as reported earlier (50).

Mice were anesthetized with xylazine-ketamine (97.5 mg/kg of body weight ketamine,12.5 mg/kg xylazine) and infected intranasally with 10^4^ PFU or 10^6^ PFU PIV5-MERS-S or PIV5-GFP in 60 μl DMEM. The mouse-adapted MERS_MA_6.1.2 strain was inactivated by exposure to UV light for 1 h using a wattage of 4,016 μW/cm^2^. Then 10^6^ PFU UV-inactivated viruses were 1:1 (vol/vol) mixed with Imject alum (Thermo, catalog no. 77161) and delivered to mice intramuscularly. Four weeks postimmunization, mice were infected intranasally with 10^5^ PFU MERS_MA_6.1.2 in 50 μl DMEM. For passive immunization, sera were collected from hDPP4 KI mice that received 10^4^ PFU PIV5-MERS-S or PIV5-GFP intranasally at 4 weeks postimmunization. Two hundred microliters of sera were transferred into hDPP4 KI mice intraperitoneally 1 day before challenge with 10^5^ PFU MERS_MA_6.1.2. Infected mice were examined daily, and weights were recorded. All work with MERS-CoV was performed in the biosafety level 3 (BSL3) laboratory of the University of Iowa.

### Histology.

At the indicated days postchallenge, mice were anesthetized and perfused with PBS by intracardiac injection followed by perfusion with zinc formalin. Lungs were removed, fixed in zinc formalin overnight, and paraffin embedded. Lung sections (∼4-μm thickness) were stained with hematoxylin and eosin. Tissues were evaluated by board-certified veterinary pathologists and scored using a postexamination masking method (51). Lungs were scored for edema, hyaline membranes, cellular debris, and hemorrhage, with scores of 0, 1, 2, 3, and 4 representing detection in 0%, less than 5%, 6% to 33%, 33% to 66%, and more than 66% of lung fields, respectively. Lungs were scored for mononuclear infiltrates, with scores of 0 representing values within normal parameters, 1 representing small aggregates in peribronchial and perivascular areas, 2 representing perivascular and periairway aggregates filling perivascular space, and 3 representing a score of 2 plus expanding sheets of infiltrates into septa and consolidation lesions in regions of the lung, respectively. Lungs were scored for granulocytic infiltrates, with scores as follows: 0, within normal parameters; 1, scattered PMNs sequestered in septa; 2, a score of 1 plus solitary PMNs extravasated in airspaces; 3, a score of 2 plus small aggregates in vessels and airspaces, respectively. Lung tissues were evaluated for perivascular eosinophil infiltration. Briefly, vessels with cellular infiltration (*n* = 20/lung) were randomly selected by a masked pathologist, and the number of eosinophils was enumerated and averaged for a final score for each lung (52).

### Neutralizing antibody assay.

Four weeks postimmunization, sera from immunized mice were collected. All serum samples were heat inactivated by incubation at 56°C for 30 min. Heat-inactivated serum was serially diluted by 2-fold in 96-well plates before the same volume of MERS-CoV pseudovirus was added and incubated at 37°C for 1 h. The mixture was added into Vero 81 cells in 96-well plates and incubated at 37°C for 1 h to allow virus binding. Then the mixture was removed, and cells were rinsed with PBS. The next day, the neutralization results were measured by luciferase assay and plotted relative to the value for serum-free wells.

### Immunoblotting and immunofluorescence.

Vero 81 cells were infected with PIV5-MERS-S at different MOIs or mock infected at 37°C for 1 h. Cell lysates were collected at 2 days postinfection and applied to Western blotting. The expression of MERS-CoV spike protein was detected by a rabbit anti-MERS-CoV S2 antibody (Sino Biological, catalog no. 40070-T60) and colocalized using a mouse anti-α-tubulin antibody (Sigma, catalog no. T9026). Indirect immunofluorescence assays were performed to detect expression of the S protein in PIV5-MERS-S-infected cells. Vero cells were infected with PIV5 or PIV5-MERS-S. Forty-eight hours later, cells were fixed with 2% formaldehyde in phosphate-buffered saline (PBS) and permeabilized by adding 0.1% saponin to the immunostaining buffers. Anti-MERS-S from Sino Biological was used (catalog no. 40070-T60). The cells were imaged using a Nikon A1R confocal microscope.

### Analyses of MERS-CoV-specific CD8 T cell response.

hDPP4 KI mice were immunized with 10^4^ PFU PIV5-GFP or PIV5-MERS-S via intranasal route. At 4 weeks postimmunization, lung cells were harvested and MERS-CoV-specific CD8^+^ T cells were stimulated with 1 μM S434 and S1165 peptides as described previously (53) in the presence of Golgi-plug (1 μl/ml) for 5 h. Cells were then washed and stained for cell surface CD45, CD4, and CD8 markers followed by intracellular IFN-gamma staining. To examine the recall CD8 T cell response, hDPP4 KI mice immunized with 10^4^ PFU PIV5-GFP or PIV5-MERS-S via intranasal route were challenged with 10^5^ PFU MERS_MA_6.1.2. Four days after MERS_MA_6.1.2 infection, lungs were harvested and CD8 T cells were stimulated with S434 and S1165 peptides in the presence of Golgi-plug (1 μl/ml) for 5 h. Cells were then washed and stained for cell surface CD45, CD4, and CD8 markers followed by intracellular IFN-gamma staining. The following monoclonal antibodies were used: PECy7 anti-CD45 (30-F11), anti-CD4 (RM4-5), and anti-CD8α (53-6.7), all from BD Bioscience, and IFN-γ (XMG1.2) from eBioscience.
